# Post-Prostatectomy Image-Guided Radiotherapy: The Invisible Target Concept

**DOI:** 10.3389/fonc.2017.00034

**Published:** 2017-03-09

**Authors:** Florent Vilotte, Mickael Antoine, Maxime Bobin, Igor Latorzeff, Stéphane Supiot, Pierre Richaud, Laurence Thomas, Nicolas Leduc, Stephane Guérif, Jone Iriondo-Alberdi, Renaud de Crevoisier, Paul Sargos

**Affiliations:** ^1^Department of Radiotherapy, Institut Bergonié, Bordeaux Cedex, France; ^2^Department of Medical Physics, Institut Bergonié, Bordeaux Cedex, France; ^3^Department of Radiotherapy, Groupe ONCORAD, Clinique Pasteur, Toulouse, France; ^4^Department of Radiotherapy, Institut de Cancérologie de L’Ouest René Gauducheau, Nantes, France; ^5^Department of Radiotherapy, CHU de Poitier, Poitiers, France; ^6^Institut Bergonié, Bordeaux Cedex, France; ^7^Department of Radiotherapy, Centre Eugène-Marquis, Rennes, France

**Keywords:** post-prostatectomy, prostate neoplasm, radiotherapy, image-guided radiotherapy, spacers, endorectal balloons, diet protocol

## Abstract

In the era of intensity-modulated radiation therapy, image-guided radiotherapy (IGRT) appears crucial to control dose delivery and to promote dose escalation while allowing healthy tissue sparing. The place of IGRT following radical prostatectomy is poorly described in the literature. This review aims to highlight some key points on the different IGRT techniques applicable to prostatic bed radiotherapy. Furthermore, methods used to evaluate target motion and to reduce planning target volume margins will also be explored.

## Introduction

Intrapelvic anatomical variations occurring between radiotherapy fractions (inter-fractions) or during the fraction (intra-fraction), corresponding to movement and/or deformation of target volumes and/or adjacent organs at risk (OAR), can result in differences between the distribution of the pretreatment-delivered dose and the initially planned distribution. If not corrected, these variations can particularly cause severe overdose to healthy tissues and underdose to target tumor, leading to an increased risk both of toxicity and of local recurrence (1). In this context, radiotherapy following radical prostatectomy represents a challenge for the radiation oncologist. The absence of a visible target within a complex pelvic anatomical region requires, firstly, accurate target volume delineation and, secondly, a qualitative approach to ensure radiation delivery.

Regarding the first condition, to date, only four articles have published consensus guidelines to delineate the clinical target volume (CTV) corresponding to the prostatectomy bed. According to the Radiation Therapy Oncology Group (RTOG) Consensus Guidelines, CTV can be defined as “the tissue volume at risk of subclinical microscopic and macroscopic tumor growth for the prostate fossa following radical prostatectomy” (2–5). Even if such definition results in lower interobserver variability in the CTV delineation (6), the characterization of the volume of interest still differs from one article to another. This uncertainty arises from both the anatomical modifications after surgery and the difficulty in using the data on preoperative target volume localization. In turn, with the development of intensity-modulated radiation therapy (IMRT), the possibility of increasing the dose to the target while sparing the surrounding OARs has led to significantly improving the biochemical control for localized prostate cancers (7–9). In the postsurgery setting, the prescribed dose has been shown to be correlated to a biochemical control with both adjuvant radiotherapy and salvage radiation therapy (10–13). The use of IMRT for the irradiation of the prostatectomy bed has also allowed reducing significantly late grade ≥2 gastrointestinal toxicity compared to 3D conformal radiation therapy (14, 15). Nevertheless, with the increase of elderly patients, the choice of treatment must be discussed, and the toxicity threshold re-defined (16). If clinical benefits appear to be obvious with IMRT, the goal of image-guided radiation therapy (IGRT) is to ensure an effective treatment delivery by precisely targeting the radiation to the tumor. The use of planning target volume (PTV), which takes into consideration the uncertainties linked to patient positioning and target volume movements and deformation during treatment, becomes a determining element to guarantee the quality of radiation therapy in the postsurgery setting (17). Dosimetry inaccuracies resulting from positioning errors may decrease biochemical control (18) and increase toxicity if the OARs are not spared (19–21). Prostatectomy bed movements and/or deformations are mainly dictated by changes in the volume and shape of rectum and bladder (18, 22). The aim of this review is to provide an overview of prostatectomy bed motion (PBM) and/or deformation in post-prostatectomy radiotherapy. Repositioning imaging techniques used in IGRT and potential corrective, preventive, and stabilizing measures will also be explored.

## Concepts and Strategy for IGRT of the Prostatectomy Bed

### What Errors Must Be Taken into Account?

Image guidance is defined as a 3D adjustment of the target position such that the treatment target and the planned target positions correspond. IGRT allows tracking the position of the patient and the target isocenter, of the PTV and of adjacent OARs, as well as analyzing possible deformations for those volumes during the radiotherapy schedule. Minimizing these repositioning errors could lead to reduced PTV margins, which facilitates OAR sparing. Positioning errors can be divided into three main categories:

– Setup errors (SUEs) correspond to the necessary displacements to align bony anatomy on the electronic portal image and the digitally reconstructed radiograph, after patient positioning using skin landmarks.
– PBM corresponds to target volume movement relative to bony anatomy.
– Total positioning error (TPE) is the sum of the two previous errors.

Both systematic (mean value of the displacement) and random (SD of the displacement) errors can be calculated, the systematic error impacting strongly in dose variations (17). In addition to displacement uncertainties, the prostatectomy bed may present large deformations, which are less observed with the intact prostate. Such anatomical variations are much more complex to quantify and to take into account than displacements, unless using elastic registration methods.

### How to Evaluate and Reduce These Errors?

The different IGRT techniques allow viewing the tumor either directly through 2D or 3D images, or indirectly using markers or bony structures closely related to the tumor and/or OAR motion. Table 1 presents potential advantages and limitations of prostatectomy bed IGRT techniques. Table 2 synthetizes the results of main IGRT studies evaluating PBM.

**Table 1:** **Description of post-prostatectomy image-guided radiotherapy (IGRT) techniques**.

IGRT technique	Concept	Advantages	Limitations
2D imaging	Displacement determined by bonny anatomy or fiducial marker misalignment between the image acquired by the treatment device compared to DRR	– Quick – Low dose	– No visualization of soft tissues
3D imaging	Image reconstructed by rotation around the patient through several 2D projections	– Alignment using skin landmarks possible – Visualization of target volume and OAR allowing to take into consideration variations due to rectal and bladder filling – Low energy (on board imaging or X-ray volume imaging)	– Artefacts related to materials with high electronic density – High energy (high-energy scan of tomotherapy devices) – Image quality
Transabdominal or transperineal ultrasound	Follow-up of target volume positioning during treatment sessions	Non-ionizing	Inter-operator variability
MRI	Treatment devices coupled to an MRI system	– Non-ionizing – Follow-up of motions during sessions – Mage quality	– Image distortion – Calculation of dose distribution
Fiducial markers	Implanted in the target volume, and theoretically follow target motion	– Account of prostatectomy bed motion contribution in case of bidimensional imaging modalities – Potential improvement in the precision of alignment using 3D imaging	Invasive procedure
Electromagnetic transponders	A real-time follow-up of transponder displacements, implanted in the target volume, allows studying intra-fraction motion	Intra-fraction and inter-fraction evaluation	Invasive procedure

*DRR, digitally reconstructed radiograph; MRI, magnetic resonance imaging; OAR, organs at risk*.

**Table 2:** **Results of IGRT main studies evaluating prostatectomy bed movements**.

Reference	IGRT technique	Patient/images	Positioning error (mean or average)	AP mm (SD)	SI mm (SD)	LR mm (SD)	Proposed PTV margins (mm)
Ost et al. (24)	CBCT	15/547	PBM mean	2.7 (3)	0.9 (1.4)	0.6 (0.9)	AP 8^c^
			PBM average	2.2	0.6	0	SI 6^c^
			TPE mean	3.1 (2.3)	1.9 (1.6)	2.9 (2.2)	LR 8^c^
			SUE mean	1.9 (1.8)	1.9 (1.5)	2.9 (2.2)	
Song et al. (32)	Surgical clips	17/364	TPE	−2.1	0.6	−0.1	AP 8^c^
	kv		Absolute shifts	3.1 (2.3)	2.5 (1.4)	2.3 (0.7)	SI 9^c^
							LR 6^c^
Sandhu et al. (31)	Surgical clips	26/692	PBM	2.7 (2.1)	2.4 (2.1)	1 (1.7)	
	kv		TPE	3.8 (5.5)	5.3 (8.1)	3.9 (5.9)	
			SUE	5.2 (7.1)	4.9 (7.5)	3.6 (5.6)	
Bell et al. (34)	Surgical clips	40/377	PBM upper	0.5 (0.5)	0.28 (0.26)	0.10 (0.12)	
	CBCT		PBM lower	0.18 (0.16)	0.18 (0.17)	0.08 (0.1)	
Huang et al. (33)	Surgical clips	14/420	PBM inter-fraction	1.9	−0.9	0	AP 4.8^a^/6.3^c^
	CBCT		PBM intra-fraction	0.2	−0.4	0.1	SI 4.6^a^/6.1^c^
							LR 3.1^a^/3.9^c^
Kupelian et al. (35)	Surgical clips	4/140	PBM	0.39 (1.27)	0.1 (0.86)	0.06 (0.37)	
	MVCT						
Ålander et al. (39)	Gold seeds	13/466	PBM	0.8 (1.6)	0.7 (2.1)	0 (0.5)	AP 6.6^b^
	CBCT		TPE	0.4 (2.7)	0.3 (2.9)	1.2 (1.8)	SI 6.5^b^
			SUE	−0.2 (2.2)	−0.5 (2)	1.2 (1.8)	LR 2.4^b^
Schiffner et al. (40)	Gold seeds	10/163	PBM	−1.1 (2.1)	0.4 (2.4)	0.3 (0.9)	
	kv (EPID)		TPE	−0.3 (4.5)	1.2 (5.1)	0.2 (4.5)	
			SUE	−0.2 (5.1)	1.1 (3.9)	0.1 (4.5)	
Klayton et al. (23)	Calypso	20/87	PBM mean	2.5 (3.2)	3.6 (4.2)	1.3 (1.8)	AP 5^a^/9^b^/15^c^
	kv		TPE mean	4 (4.9)	3.8 (5.2)	3 (4.1)	SI 5^a^/13^b^/13^c^
			SUE mean	4.1 (4.7)	4.1 (5.2)	3.9 (5.2)	LR 5^a^/5^b^/9^c^
			PTV-CTVm1	9	13	5	
Cavalieri et al. (36)	CT on rail	17/661	TPE mean	4.7 (3.3)	3.8 (3.0)	2.9 (2.5)	
			TPE average	−2.2 (5.3)	−1.1 (4.7)	−0.6 (3.8)	
Simpson et al. (25)	CBCT	23/585	PBM (CBCT)	0.9 (1.6)	0.5 (1.5)	0.4 (0.9)	
	kv						

*Margin recipe used: 2.5Σ + 0.7σ and 1.96Σ + 0.7σ*.

*^a^PTV-CTV margins calculated with respect to PBM (using IGRT technique analyzed in the study)*.

*^b^PTV-CTV margins calculated with respect to TPE (verification of bony anatomy alignment)*.

*^c^PTV-CTV margins calculated in case of absence of IGRT*.

*PBM, prostatectomy bed motion; SUE, setup error; TPE, total positioning error; AP, anteroposterior; SI, superoinferior; LR, left–right; CTV, clinical target volume; PTV, planning target volume; IGRT, image-guided radiotherapy; Surg. Clips, surgical clips; CBCT, cone beam computed tomography; MVCT, megavoltage computed tomography; kv, kilovoltage; EPID, electronic portal imaging device; CT, computed tomography*.

## Image-Based Positioning Techniques in Post-Prostatectomy IGRT: What Results with Which Technique?

### Bony Anatomy Alignment Captured by 2D Imaging

Klayton et al. studied PBM using electromagnetic transponders in order to evaluate the quality of bony anatomy as a localization method using 2D imaging. After patient positioning based on laser and skin landmarks, the evaluation of target volume isocenter position was carried out with electromagnetic transponders. Deviation of the isocenter position in this case corresponded to TPE. Once the first alignment completed, 2D kv–kv imaging was performed. PBM was estimated by measuring the 3D shifts needed to align bony anatomy. For 9% of fractions, anterior–posterior (AP) direction PBM exceeded 5 mm. In 21% of fractions, a repositioning in the superior–inferior (SI) direction was necessary. Finally, 70% of patients were repositioned at least once during treatment. According to the authors, patient setup margins were 5 mm in left–right (LR), 13 mm in SI, and 9 mm in AP based on 2D kv–kv image guidance. The results of this study are summarized in Table 2. 2D imaging on its own only takes into consideration SUEs, omitting the contribution of the PBM component and of volume variations. As a result, it is not adapted to estimate prostatectomy bed movements (23).

### Soft Tissue Anatomy Alignment Evaluated by 3D Imaging

Ost et al. analyzed a series of 547 cone beam computed tomography (CBCT) daily images from 15 patients successively treated by post-prostatectomy radiotherapy. PBM was determined considering the motion of the anterior rectal wall. Systematic inter-fraction movements in the LR, SI, and AP were 0.44, 0.92, and 2.50 mm, respectively. Similarly, random deviations of 0.99 mm in LR, 1.38 mm in SI, and 2.32 mm in AP axes were observed. These results, based on imaging modalities that take into account PBM, emphasize the prevalence of AP shifts of the prostatectomy bed, as reported for prostate, and highlight that an approach relying only on bony anatomy appears insufficient (24). Despite the larger TPE described with 2D kv–kv imaging compared to CBCT, no correlation was found between TPE and acute toxicity (25). Using computed tomography (CT)-on-rails IGRT on 10 patients, Liu et al. analyzed volume variations and deformations of CTV, rectum, and bladder. They showed daily volume variations of 75–116% for CTV, 50–270% for rectum, and 30–180% for bladder compared to planning CT (26).

### Soft Tissue Anatomy Alignment Evaluated by Ultrasonography (US)

Studies on the use of US for post-prostatectomy IGRT are scarce. Chinnaiyan et al. analyzed PBM in six consecutive patients by comparing transabdominal US (taking the bladder neck as reference for post-prostatectomy fossa localization) with 2D imaging. Regarding repositioning accuracy, there was a difference of 5 ± 3 mm between the two techniques in favor of US imaging. This result supports the use of the US-IGRT for daily pretreatment patient repositioning as stated by the authors (27).

A comparison of transabdominal US and CBCT imaging was carried out by Fargier-Voiron et al. in 11 post-prostatectomy patients. The differences between US and CBCT shifts were −0.7 ± 4.3, 1 ± 4.6, and 0.2 ± 2.7 mm in AP, SI, and LR axes, respectively. For these three directions, the shift agreements (percentage of sessions for which the shift difference between the two modalities is below or equal to 5 mm) between US and CBCT were 80.2, 86.8, and 96.2%, respectively. During radiotherapy schedule, 20% of the US images were excluded due to poor quality, the authors concluding that transabdominal US imaging alone should not be used as IGRT modality (28). The same group evaluated a novel method of transperineal US imaging (Clarity, Elekta^®^) that offers a better image quality (100 vs 80% exploitable), a reduction of inter-operator variability, and a consistent probe pressure during examination. Shift agreements at ±5 mm improved to 90.3, 85, and 97.6% in AP, SI, and LR directions, respectively, leading the authors to propose this method as a non-ionizing alternative to CBCT (29).

### What PTV Margins Are Used with Which IGRT Technique?

It appears essential to adapt PTV margins to the IGRT techniques used by the physician. According to the literature, these margins range from 3 to 10 mm (30). For example, in cases of bony anatomy alignment, PTV margins vary from 5 to 15 mm. Indeed, recommendations for target volume definitions differ substantially: at least 5 mm according to the European Organization For Research and Treatment of Cancer, 10 mm according to the Australian and New Zealand Radiation Oncology Genito-Urinary Group (in case of rectal dose–volume histogram limitations, a reduction of the posterior PTV margin expansion to 5 mm is possible), from 6 to 15 mm according to the RTOG study 0534, and from 5 to 15 mm according to the recommendations from the Groupe d’Etude des Tumeurs Uro-Génitales (2–5).

## Can IGRT be Improved Using Prostatectomy Bed Repositioning Markers?

### Surgical Clips As Markers

**Figure 1:**
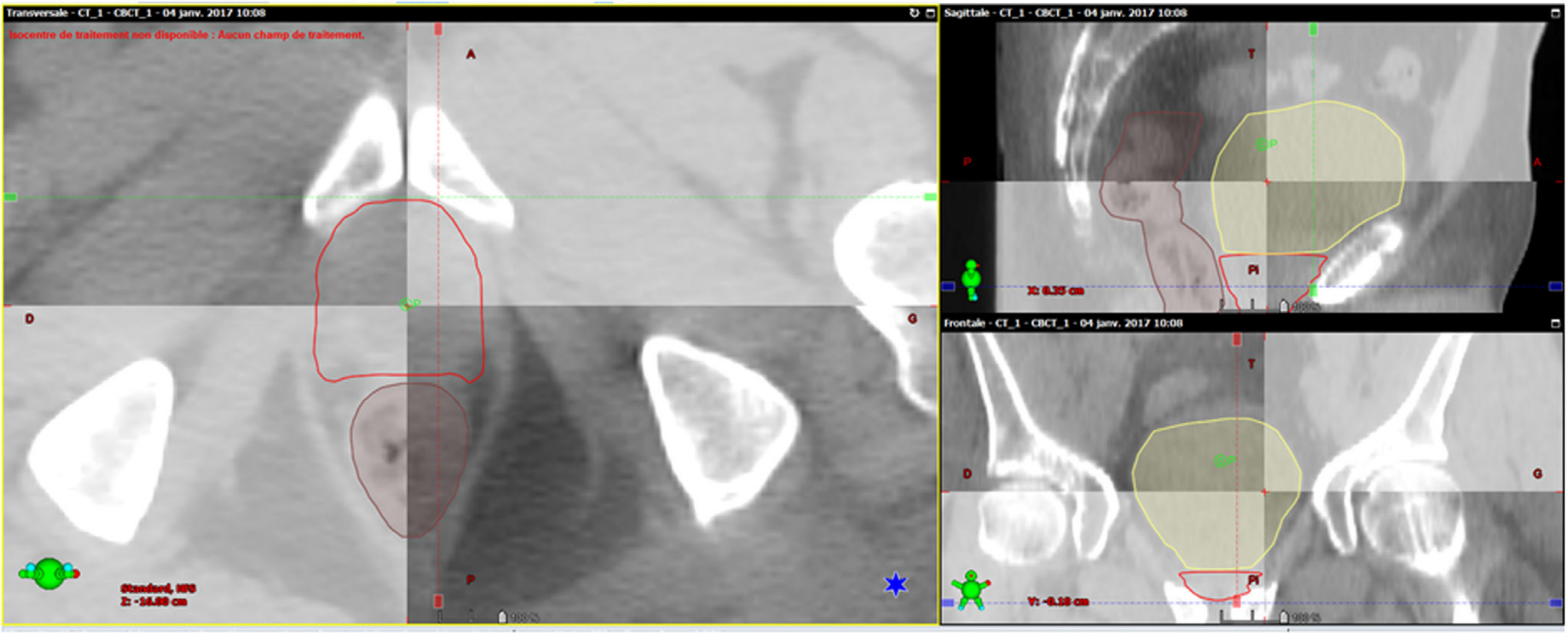
**Example of post-prostatectomy image-guided radiotherapy**. Initial computed tomography (CT) scan and cone beam computed tomography (CBCT) used for analysis of repositioning show a good correlation for rectum, bladder, and clinical target volume.

Similar to prostate radiotherapy, several studies have analyzed the use of surgical clips during post-prostatectomy irradiation (31–35). Sandhu et al. studied two orthogonal kv images to localize the prostatectomy bed in 26 patients using surgical clips as target volume landmarks. In total, 692 images were analyzed. Target volume displacements were mainly related to SUE. PBM was most prominent in the AP axis, with an average magnitude of 2.7 ± 2.1 mm. PBM in the SI and LR directions was 2.4 ± 2.1 and 1 ± 1.7 mm, respectively (31). Series using 3D imaging with the same approach have also reported the prominence of the displacements in the AP direction (2–5 mm) compared to those in the SI (0.5–2.5 mm) and lateral (less than 1 mm) directions (33, 35). Cavalieri et al. used surgical clips as markers to analyze the repositioning of 17 consecutive patients using CT-on-rail IGRT. Systematic errors led to displacements ranging from 6 to 10 mm, mainly in the AP dimension (5.5%) (36). Hence, the use of surgical clips as markers to guide radiotherapy could reduce the impact of PBM. Figure 1 presents an example of CBCT for post-prostatectomy radiotherapy.

### Gold Fiducial As Markers

Historically used for prostate radiotherapy, fiducial markers facilitate the detection of the CTV. Additionally, their radiopacity allows using low ionizing imaging modalities. Even though the transrectal implantation of gold fiducial markers under US guidance is an invasive technique, very few complications have been described. Langenhuijsen et al. implanted three gold markers (two at the dorsal bladder base and one next to the anastomosis) in 77 consecutive post-prostatectomy patients and showed the feasibility of the procedure (37). Fortin et al. reported a reduction of inter-operator variability during online and offline localization compared to surgical clips. Furthermore, fiducial markers resulted in a repositioning quality greater than that of surgical clips, often out of the prostatectomy bed and closely clustered, limiting daily SUE and errors related to PBM (38). After implanting gold fiducial markers into the prostate bed, daily CBCT of 13 patients was analyzed by Ålander et al. They reported displacements (mean ± SD) of 0.0 ± 0.5 mm in the LR, 0.7 ± 2.1 mm in the SI, and 0.8 ± 1.6 mm in the AP directions, which were deemed non-significant by the authors (39). In a similar manner, Schiffner et al. used 2D imaging for 10 patients. Positioning errors of more than 5 mm in the LR, SI, and AP axes were observed in 14.1, 38.7, and 28.2% of the cases, respectively, mainly related to SUE, while PBM remained modest. Over the total duration of treatment, gold seed fiducial migration was small (0.4 mm on average) (40). Confirming the difficulties in matching predominant in the AP direction, also reported with the use of surgical clips, gold fiducial markers with CBCT or kv–kv imaging appear to be more robust despite their invasive nature.

## Is There a Need for Global Positioning System for Prostate Bed Radiotherapy?

The Calypso^®^ 4D Localization System commercialized by Varian enables real-time intra-fraction localization and tracking with three electromagnetic transponders (41). Already studied in the prostate irradiation setting (42), intra-fraction motion was analyzed in 20 patients undergoing post-prostatectomy radiotherapy. A displacement of more than 5 mm during 30 s was reported for at least 11% of delivered fractions. For 16 (80%) patients, PBM was observed in the SI and AP axes, and the 5-mm threshold margin was exceeded in a third of cases. Interruptions for repositioning were reported in 15% of the delivered fractions. Over the treatment course, only 25% of the patients were repositioned more than five times, and 30% of the patients did not need any repositioning. Further studies are needed in order to select patients that can benefit most from this approach (23).

## Preventive, Corrective, and Stabilizing Approaches to Limit PBM Due to Rectal and Bladder Movements

**Figure 2:**
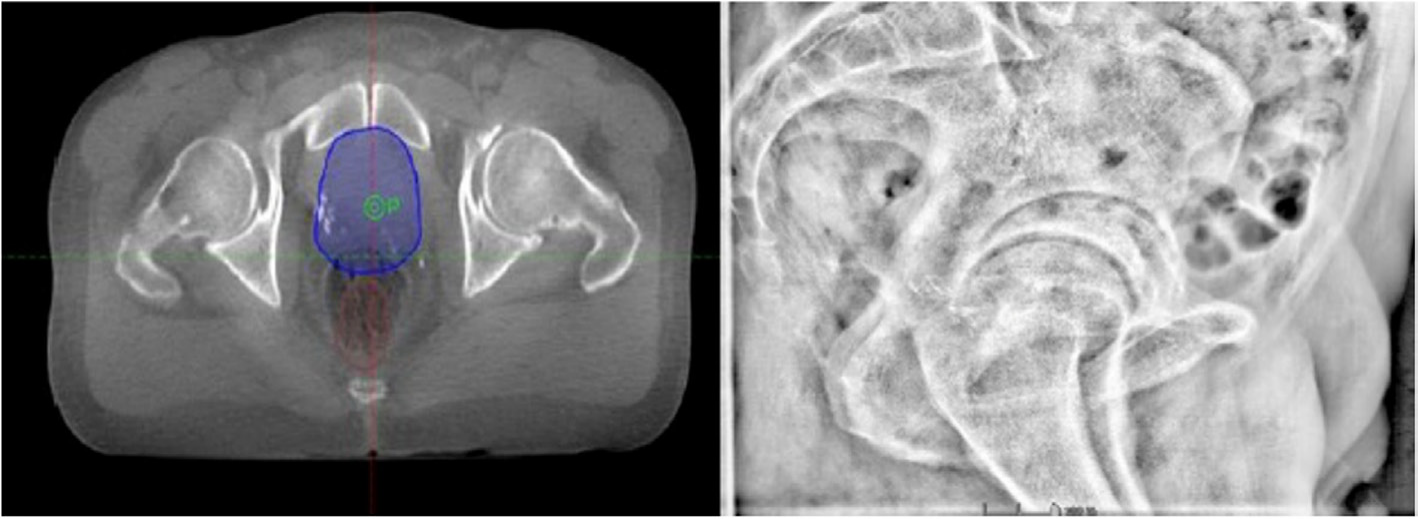
**Example of a patient treated by prostate-bed radiotherapy with inadequate rectal filling during image-guided radiotherapy (3D image-based positioning)**.

Prostatectomy bed motion is essentially correlated with adjacent OAR displacements or volume variations. Disregarding rectal distension could result in an increase of up to 18 or 24 mm of posterior margins and, consequently, in dosimetry inaccuracies (43, 44). Figure 2 illustrates a case of inadequate rectal filling during treatment. Concerning bladder volume variations during treatment, Fiorino et al. detected a ratio between the largest and smallest volume of 3.8 (range 1.9–8.3), which had an impact on PTV (18). Preventive, corrective, and stabilizing approaches to limit PBM due to rectal and bladder movements are presented below.

### Bladder Filling

Variations in bladder volume are frequent in both post-prostatectomy and non-operated patients, with a trend in decreasing volume during treatment (18, 45). These bladder volume variations could impact on PTV coverage (22). Bell et al. showed that bladder filling variations of >2, ±1, or <2 cm happened in 3.4–56.2% of cases, with most size changes occurring in the AP direction. These variations resulted in potential geographic misses (movement of surgical clips greater than 0.5 cm posteriorly, 1 cm in other directions). Further, if the bladder or rectum remained within 1 cm of the planned size, less than 10% of the images revealed a geographic miss (46). These results demonstrate the importance of a consistent and stable bladder and rectal volume during treatment. Patients at risk of variations should be detected early in order to offer them the most secure treatment.

### Diet Recommendations

Studies on diet changes, in order to prevent the production of gas, primarily in cases of prostate irradiation, have led to conflicting results. Smitsmans et al. compared the CBCT of 26 patients irradiated following a diet poor in fibers and 23 patients following no diet. Diet was beneficial, preventing the presence of stool, gas pockets, and moving gas pockets during radiotherapy, and resulted in a reduction of inter-fraction and intra-fraction motion (47). On the contrary, Lips et al. evaluated the same type of diet in 105 patients and observed an increase in inter-fraction prostate motion for patients following the dietary protocol. The median of the average inter-fraction motion ranged from 2.53 mm in the non-diet group to 3 mm in the diet group (48). Using magnetic resonance imaging (MRI) as IGRT technique, Nichol et al. evaluated an antiflatulent diet and a milk of magnesia-based laxative in 42 patients and did not observe a reduction in inter-fraction prostate motion. This study demonstrated that moving gas only (56%) and moving gas and stool (18%) accounted for 74% of inter-fraction movements (49). Concerning inter-fraction motion, Oates et al. could not demonstrate any significant difference in favor of a diet associated to psyllium but observed a trend toward rectal volume reduction (50). A randomized trial including 40 patients studied the use of probiotics, such as *Lactobacillus acidophilus*, and reported a significant reduction in rectal volume variations over the treatment course (51). Evaluations on other molecules preventing the production of intestinal gas, such as the alpha-galactosidase, are ongoing (52). Although encouraging, these results need to be further validated.

### Strategies for Rectal Emptying

Several studies have analyzed the efficacy of different strategies to empty rectal gases in order to minimize prostate motion. Yahya et al. compared three strategies to reduce rectal distension: (i) the use of microenema before each treatment; (ii) a recommended dietary protocol; and (iii) no bowel preparation or dietary advice. After the analyses of the CBCT scans of these three groups, a reduction of almost half of scans showing geometric miss (shifts ≥5 mm) was observed in the microenema group compared to the other two (53). Ogino et al. analyzed the impact of rectal gas self-evacuation using the index finger on the average prostate and seminal vesicle motion in 76 patients. A significant reduction (0.3–4.4 mm) of the prostate and the seminal vesicle displacement was observed (54). Diot et al. analyzed in a series of 17 post-prostatectomy patients an intervention involving the use of a rectal catheter to deflate the rectum, evacuation of stools, and adjustment of bladder filling. These corrective measures were applied in cases of rectal or bladder wall displacements larger than 5 mm. The median number of interventions per patient was 5. The procedure led to a reduction in the motion of the target volume during radiotherapy schedule, which dropped from 45 to 21% in the AP, from 7 to 4% in the SI, and from 7 to 8% in the LR direction. These measures, more effective for AP displacements, decreased the PTV margin by 3.3 mm (55). Nevertheless, no benefit in terms of dosimetry was observed with conventional fractionation both for PTV coverage or OAR sparing. These results suggest that daily CBCT localization alone could be enough to take into consideration the motion of the target volume. For hypofractionated treatments, however, the rectal emptying interventions could have a greater impact in terms of dosimetry (56).

### Endorectal Balloons (ERBs)

Rectal filling has been identified as predictive of prostate motion by cine-MRI studies assessing intra-fraction movements (57, 58). The introduction of an ERB could optimize the rectal volume and conformation, minimizing at the same time target volume positioning errors.

In a comparative study on 14 post-prostatectomy patients, 7 of which were treated with ERB, shift agreement of CTV and rectal volumes with planning CT were improved by 4 and 21%, respectively. This stability is also reflected in a reduction of median motion, particularly the AP margin of the lower part of the CTV motion of 0.43 ± 0.45 cm without ERB to 0.37 ± 0.27 cm with ERB. The lower part of CTV moves dropped from 0.16 ± 0.17 to 0.11 ± 0.11 cm. ERB also reduced the impact of vesicle filling on shift agreement (59). Jameson et al. analyzed the use of ERB on post-prostatectomy patients and observed no significant dosimetry improvements in terms of PTV coverage or OAR sparing with the use of ERB (60). On the other hand, some dosimetry studies have demonstrated an improvement in rectum and anal canal sparing, mainly for intermediate and high doses, with the use of ERB. Smeenk et al. compared the dosimetry in 20 patients that had undergone surgery, with or without ERB, for a prescription dose of 70 Gy. Regarding rectal dose–volume histogram, rectal V30 and V40 dropped by 8 and 5%, respectively. CTV volume was considerably reduced in the presence of ERB (117 ± 27 vs 110 ± 20 cc), but no correlation could be found between this volume and rectal sparing (61, 62).

Concerning the clinical impact, Ishiyama et al. carried out a retrospective study on 107 patients treated by salvage radiotherapy with ERB at a dose of 70 Gy in 32 fractions. Late gastrointestinal and genitourinary toxicities of grade 2 were reported in 6 and 13% of patients, respectively, and grade 3 in 3 and 6% of patients, respectively (63).

The development of new stabilizers, such as the ProSpare of the Institute of Cancer Research in London, opens up a new study field in this domain. ProSpare proposes to add radio-opaque markers that allow a better identification of the anterior rectal wall, as well as ventilation holes for the evacuation of rectal gases (64, 65). A phase II study is currently ongoing (postoperative ProSpare).

### Spacers

The injection or implantation of a biodegradable substance in the anterior perirectal fatty space was studied for patients receiving prostate radiotherapy (66). This approach allows displacing the prostate away from the rectal wall reducing the rectal volume exposed to high level doses (67). Pinkawa et al. found a significant reduction of systematic posterior displacements superior to 6.5 mm (dropping from 27 to 0%) (68–71). For prostate radiotherapy, a wide range of spacers have been studied, and the prostate-rectum separation varied from 7 to 20 mm depending on the technique used, reducing the rectal V70 by about 43–84% (66, 72, 73). Spacer utilization has been less explored in the postoperative radiotherapy setting. Pinkawa et al. published a case report on a patient presenting a macroscopic recurrence at the uretho-vesical anastomosis. Polyethylene glycol spacer injection allowed them to create a space of more than 1 cm between the recurrence site and the rectal wall. This led to significantly reducing the rectal V70, V60, and V50 compared to treatment planning based on computer tomography. PTV dose prescription was 76 Gy, and a good global tolerance led the authors to propose this approach for specifically selected patients (74).

## Conclusion

Radiotherapy of the prostatectomy bed in an adjuvant or salvage setting, mostly under IMRT and IGRT conditions, constitutes a routine situation for the clinician. 2D imaging modalities are in themselves insufficient to evaluate target volume displacement and deformation, and the soft tissue anatomy alignment using a 3D approach appears crucial. The utility of fiducial markers or surgical clips, as well as preventive, corrective, or stabilizing measures, has been shown to limit these displacements. At present, due to lack of substantial literature, reducing the margin that constitutes the PTV to less than 5 mm (independent of the IGRT technique used) is not recommended; however, an anisotropic approach can be justified in view of the predominant displacements in the AP dimension on the prostatectomy bed. The development of MRI and of tracking strategies could therefore improve imaging quality and, as a result, increase the precision of soft tissue anatomy alignment. The trend toward dose augmentation and hypofractionation requires not only precise target localization to ensure dose distribution but also tolerance and efficacy. Confirming the impact of IGRT by means of larger studies seems necessary with, notably, an evaluation of patient-reported outcomes.

## Author Contributions

Drafting the article: FV and PS. Critical revision of the article; final approval of the version to be published: FV, MA, MB, IL, SS, PR, LT, NL, SG, JI-A, RC, and PS.

## Conflict of Interest Statement

The authors declare that the research was conducted in the absence of any commercial or financial relationships that could be construed as a potential conflict of interest.

## References

[1] Gill S Isiah R Adams R Dang K Siva S Tai KH Conventional margins not sufficient for post-prostatectomy prostate bed coverage: an analysis of 477 cone-beam computed tomography scans. Radiother Oncol (2014) 110(2):235–9. 10.1016/j.radonc.2013.12.004 24485766

[2] Poortmans P Bossi A Vandeputte K Bosset M Miralbell R Maingon P Guidelines for target volume definition in post-operative radiotherapy for prostate cancer, on behalf of the EORTC Radiation Oncology Group. Radiother Oncol (2007) 84(2):121–7. 10.1016/j.radonc.2007.07.017 17706307

[3] Wiltshire KL Brock KK Haider MA Zwahlen D Kong V Chan E Anatomic boundaries of the clinical target volume (prostate bed) after radical prostatectomy. Int J Radiat Oncol Biol Phys (2007) 69(4):1090–9. 10.1016/j.ijrobp.2007.04.068 17967303

[4] Michalski JM Lawton C El Naqa I Ritter M O’Meara E Seider MJ Development of RTOG consensus guidelines for the definition of the clinical target volume for postoperative conformal radiation therapy for prostate cancer. Int J Radiat Oncol Biol Phys (2010) 76(2):361–8. 10.1016/j.ijrobp.2009.02.006 19394158 PMC2847420

[5] Sidhom MA Kneebone AB Lehman M Wiltshire KL Millar JL Mukherjee RK Post-prostatectomy radiation therapy: consensus guidelines of the Australian and New Zealand Radiation Oncology Genito-Urinary Group. Radiother Oncol (2008) 88(1):10–9. 10.1016/j.radonc.2008.05.006 18514340

[6] Mitchell DM Perry L Smith S Elliott T Wylie JP Cowan RA Assessing the effect of a contouring protocol on postprostatectomy radiotherapy clinical target volumes and interphysician variation. Int J Radiat Oncol Biol Phys (2009) 75(4):990–3. 10.1016/j.ijrobp.2008.12.042 19345515

[7] Kupelian PA Ciezki J Reddy CA Klein EA Mahadevan A. Effect of increasing radiation doses on local and distant failures in patients with localized prostate cancer. Int J Radiat Oncol Biol Phys (2008) 71(1):16–22. 10.1016/j.ijrobp.2007.09.020 17996382

[8] Hummel S Simpson EL Hemingway P Stevenson MD Rees A Intensity-modulated radiotherapy for the treatment of prostate cancer: a systematic review and economic evaluation. Health Technol Assess (2010) 14(47):1–108, iii–iv. 10.3310/hta14470 21029717

[9] Alicikus ZA Yamada Y Zhang Z Pei X Hunt M Kollmeier M Ten-year outcomes of high-dose, intensity-modulated radiotherapy for localized prostate cancer. Cancer (2011) 117(7):1429–37. 10.1002/cncr.25467 21425143

[10] Bernard JR Buskirk SJ Heckman MG Diehl NN Ko SJ Macdonald OK Salvage radiotherapy for rising prostate-specific antigen levels after radical prostatectomy for prostate cancer: dose-response analysis. Int J Radiat Oncol Biol Phys (2010) 76(3):735–40. 10.1016/j.ijrobp.2009.02.049 19464818

[11] King CR The dose-response of salvage radiotherapy following radical prostatectomy: a systematic review and meta-analysis. Radiother Oncol (2016) 121(2):199–203. 10.1016/j.radonc.2016.10.026 27863963

[12] Herrera FG Berthold DR. Radiation therapy after radical prostatectomy: implications for clinicians. Front Oncol (2016) 6:117. 10.3389/fonc.2016.00117 27242957 PMC4860423

[13] Beck M Barelkowski T Kaul D Wecker S Thieme AH Zwahlen DR Role of dose intensification for salvage radiation therapy after radical prostatectomy. Front Oncol (2016) 6:48. 10.3389/fonc.2016.00048 26973815 PMC4771737

[14] Goenka A Magsanoc JM Pei X Schechter M Kollmeier M Cox B Improved toxicity profile following high-dose postprostatectomy salvage radiation therapy with intensity-modulated radiation therapy. Eur Urol (2011) 60(6):1142–8. 10.1016/j.eururo.2011.08.006 21855208

[15] Raziee H Berlin A. Gaps between evidence and practice in postoperative radiotherapy for prostate cancer: focus on toxicities and the effects on health-related quality of life. Front Oncol (2016) 6:70. 10.3389/fonc.2016.00070 27047800 PMC4805642

[16] Goineau A d’Aillières B de Decker L Supiot S. Integrating geriatric assessment into decision-making after prostatectomy: adjuvant radiotherapy, salvage radiotherapy, or none? Front Oncol (2015) 5:227. 10.3389/fonc.2015.00227 26528437 PMC4606064

[17] van Herk M Remeijer P Lebesque JV. Inclusion of geometric uncertainties in treatment plan evaluation. Int J Radiat Oncol (2002) 52(5):1407–22. 10.1016/S0360-3016(01)02805-X 11955756

[18] Fiorino C Foppiano F Franzone P Broggi S Castellone P Marcenaro M Rectal and bladder motion during conformal radiotherapy after radical prostatectomy. Radiother Oncol (2005) 74(2):187–95. 10.1016/j.radonc.2004.10.002 15734207

[19] de Crevoisier R Tucker SL Dong L Mohan R Cheung R Cox JD Increased risk of biochemical and local failure in patients with distended rectum on the planning CT for prostate cancer radiotherapy. Int J Radiat Oncol Biol Phys (2005) 62(4):965–73. 10.1016/j.ijrobp.2004.11.032 15989996

[20] Potters L Gaspar LE Kavanagh B Galvin JM Hartford AC Hevezi JM American Society for Therapeutic Radiology and Oncology (ASTRO) and American College of Radiology (ACR) practice guidelines for image-guided radiation therapy (IGRT). Int J Radiat Oncol Biol Phys (2010) 76(2):319–25. 10.1016/j.ijrobp.2009.09.042 20117284

[21] Korreman S Rasch C McNair H Verellen D Oelfke U Maingon P The European Society of Therapeutic Radiology and Oncology-European Institute of Radiotherapy (ESTRO-EIR) report on 3D CT-based in-room image guidance systems: a practical and technical review and guide. Radiother Oncol (2010) 94(2):129–44. 10.1016/j.radonc.2010.01.004 20153908

[22] Haworth A Paneghel A Herschtal A Duchesne G Williams S Tai KH Verification of target position in the post-prostatectomy cancer patient using cone beam CT. J Med Imaging Radiat Oncol (2009) 53(2):212–20. 10.1111/j.1754-9485.2009.02057.x 19527370

[23] Klayton T Price R Buyyounouski MK Sobczak M Greenberg R Li J Prostate bed motion during intensity-modulated radiotherapy treatment. Int J Radiat Oncol Biol Phys (2012) 84(1):130–6. 10.1016/j.ijrobp.2011.11.041 22330987 PMC3285397

[24] Ost P De Meerleer G De Gersem W Impens A De Neve W. Analysis of prostate bed motion using daily cone-beam computed tomography during postprostatectomy radiotherapy. Int J Radiat Oncol Biol Phys (2011) 79(1):188–94. 10.1016/j.ijrobp.2009.10.029 20378272

[25] Simpson DR Einck JP Nath SK Sethi RA Wang JZ Mundt AJ Comparison of daily cone-beam computed tomography and kilovoltage planar imaging for target localization in prostate cancer patients following radical prostatectomy. Pract Radiat Oncol (2011) 1(3):156–62. 10.1016/j.prro.2010.12.002 24673945

[26] Liu F Ahunbay E Lawton C Li XA. Assessment and management of interfractional variations in daily diagnostic-quality-CT guided prostate-bed irradiation after prostatectomy. Med Phys (2014) 41(3):031710. 10.1118/1.4866222 24593714

[27] Chinnaiyan P Tomée W Patel R Chappell R Ritter M. 3D-ultrasound guided radiation therapy in the post-prostatectomy setting. Technol Cancer Res Treat (2003) 2(5):455–8. 10.1177/153303460300200511 14529311

[28] Fargier-Voiron M Presles B Pommier P Munoz A Rit S Sarrut D Ultrasound versus Cone-beam CT image-guided radiotherapy for prostate and post-prostatectomy pretreatment localization. Phys Med (2015) 31(8):997–1004. 10.1016/j.ejmp.2015.07.147 26422200

[29] Fargier-Voiron M Presles B Pommier P Munoz A Rit S Sarrut D Evaluation of a new transperineal ultrasound probe for inter-fraction image-guidance for definitive and post-operative prostate cancer radiotherapy. Phys Med (2016) 32(3):499–505. 10.1016/j.ejmp.2016.01.481 26851164

[30] Ramiandrisoa F Duvergé L Castelli J Nguyen TD Servagi-Vernat S de Crevoisier R. [Clinical to planning target volume margins in prostate cancer radiotherapy]. Cancer Radiother (2016) 20(6–7):629–39. 10.1016/j.canrad.2016.07.095 27614515

[31] Sandhu A Sethi R Rice R Wang J-Z Marcus L Salem C Prostate bed localization with image-guided approach using on-board imaging: reporting acute toxicity and implications for radiation therapy planning following prostatectomy. Radiother Oncol (2008) 88(1):20–5. 10.1016/j.radonc.2008.05.009 18524399

[32] Song S Yenice KM Kopec M Liauw SL. Image-guided radiotherapy using surgical clips as fiducial markers after prostatectomy: a report of total setup error, required PTV expansion, and dosimetric implications. Radiother Oncol (2012) 103(2):270–4. 10.1016/j.radonc.2011.07.024 21890224

[33] Huang K Palma DA Scott D McGregor D Gaede S Yartsev S Inter- and intrafraction uncertainty in prostate bed image-guided radiotherapy. Int J Radiat Oncol Biol Phys (2012) 84(2):402–7. 10.1016/j.ijrobp.2011.12.035 22381905

[34] Bell LJ Cox J Eade T Rinks M Kneebone A. Prostate bed motion may cause geographic miss in post-prostatectomy image-guided intensity-modulated radiotherapy. J Med Imaging Radiat Oncol (2013) 57(6):725–32. 10.1111/1754-9485.12089 24283563

[35] Kupelian PA Langen KM Willoughby TR Wagner TH Zeidan OA Meeks SL. Daily variations in the position of the prostate bed in patients with prostate cancer receiving postoperative external beam radiation therapy. Int J Radiat Oncol Biol Phys (2006) 66(2):593–6. 10.1016/j.ijrobp.2006.05.071 16966001

[36] Cavalieri R Gay HA Liu J Ferreira MC Mota HC Sibata CH Total error shift patterns for daily CT on rails image-guided radiotherapy to the prostate bed. Radiat Oncol (2011) 6:142. 10.1186/1748-717X-6-142 22024279 PMC3220642

[37] Langenhuijsen JF Donker R McColl GM Kiemeney LA Witjes JA van Lin EN. Postprostatectomy ultrasound-guided transrectal implantation of gold markers for external beam radiotherapy. Technique and complications rate. Strahlenther Onkol (2013) 189(6):476–81. 10.1007/s00066-013-0323-4 23604186

[38] Fortin I Carrier J-F Beauchemin M-C Béliveau-Nadeau D Delouya G Taussky D. Using fiducial markers in the prostate bed in postprostatectomy external beam radiation therapy improves accuracy over surgical clips. Strahlenther Onkol (2014) 190(5):46771. 10.1007/s00066-014-0631-3 24557058

[39] Ålander E Visapää H Kouri M Keyriläinen J Saarilahti K Tenhunen M. Gold seed fiducials in analysis of linear and rotational displacement of the prostate bed. Radiother Oncol (2014) 110(2):256–60. 10.1016/j.radonc.2013.10.037 24332022

[40] Schiffner DC Gottschalk AR Lometti M Aubin M Pouliot J Speight J Daily electronic portal imaging of implanted gold seed fiducials in patients undergoing radiotherapy after radical prostatectomy. Int J Radiat Oncol Biol Phys (2007) 67(2):610–9. 10.1016/j.ijrobp.2006.09.042 17236978

[41] Balter JM Wright JN Newell LJ Friemel B Dimmer S Cheng Y Accuracy of a wireless localization system for radiotherapy. Int J Radiat Oncol Biol Phys (2005) 61(3):933–7. 10.1016/j.ijrobp.2004.11.009 15708277

[42] Willoughby TR Kupelian PA Pouliot J Shinohara K Aubin M Roach M Target localization and real-time tracking using the Calypso 4D localization system in patients with localized prostate cancer. Int J Radiat Oncol Biol Phys (2006) 65(2):528–34. 10.1016/j.ijrobp.2006.01.050 16690435

[43] Li XA Yu C Holmes T. A systematic evaluation of air cavity dose perturbation in megavoltage x-ray beams. Med Phys (2000) 27(5):1011–7. 10.1118/1.598966 10841404

[44] Pinkawa M Siluschek J Gagel B Demirel C Asadpour B Holy R Influence of the initial rectal distension on posterior margins in primary and postoperative radiotherapy for prostate cancer. Radiother Oncol (2006) 81(3):284–90. 10.1016/j.radonc.2006.10.028 17125866

[45] O’Doherty UM McNair HA Norman AR Miles E Hooper S Davies M Variability of bladder filling in patients receiving radical radiotherapy to the prostate. Radiother Oncol (2006) 79(3):335–40. 10.1016/j.radonc.2006.05.007 16781790

[46] Bell LJ Cox J Eade T Rinks M Kneebone A. The impact of rectal and bladder variability on target coverage during post-prostatectomy intensity modulated radiotherapy. Radiother Oncol (2014) 110(2):245–50. 10.1016/j.radonc.2013.10.042 24560757

[47] Smitsmans MHP Pos FJ de Bois J Heemsbergen WD Sonke J-J Lebesque JV The influence of a dietary protocol on cone beam CT-guided radiotherapy for prostate cancer patients. Int J Radiat Oncol Biol Phys (2008) 71(4):1279–86. 10.1016/j.ijrobp.2008.03.036 18572088

[48] Lips IM Kotte ANTJ van Gils CH van Leerdam ME van der Heide UA van Vulpen M. Influence of antiflatulent dietary advice on intrafraction motion for prostate cancer radiotherapy. Int J Radiat Oncol Biol Phys (2011) 81(4):e401–6. 10.1016/j.ijrobp.2011.04.062 21664067

[49] Nichol AM Warde PR Lockwood GA Kirilova AK Bayley A Bristow R A cinematic magnetic resonance imaging study of milk of magnesia laxative and an antiflatulent diet to reduce intrafraction prostate motion. Int J Radiat Oncol Biol Phys (2010) 77(4):1072–8. 10.1016/j.ijrobp.2009.06.005 19783378

[50] Oates RW Schneider ME Lim Joon M McPhee NJ Jones DK Foroudi F A randomised study of a diet intervention to maintain consistent rectal volume for patients receiving radical radiotherapy to the prostate. Acta Oncol (2014) 53(4):569–71. 10.3109/0284186X.2013.854927 24237391

[51] Ki Y Kim W Nam J Kim D Lee J Park D Probiotics for rectal volume variation during radiation therapy for prostate cancer. Int J Radiat Oncol Biol Phys (2013) 87(4):646–50. 10.1016/j.ijrobp.2013.07.038 24054874

[52] Di Stefano M Miceli E Gotti S Missanelli A Mazzocchi S Corazza GR. The effect of oral alpha-galactosidase on intestinal gas production and gas-related symptoms. Dig Dis Sci (2007) 52(1):78–83. 10.1007/s10620-006-9296-9 17151807

[53] Yahya S Zarkar A Southgate E Nightingale P Webster G. Which bowel preparation is best? Comparison of a high-fibre diet leaflet, daily microenema and no preparation in prostate cancer patients treated with radical radiotherapy to assess the effect on planned target volume shifts due to rectal distension. Br J Radiol (2013) 86(1031):20130457. 10.1259/bjr.20130457 23995876 PMC3830438

[54] Ogino I Uemura H Inoue T Kubota Y Nomura K Okamoto N. Reduction of prostate motion by removal of gas in rectum during radiotherapy. Int J Radiat Oncol Biol Phys (2008) 72(2):456–66. 10.1016/j.ijrobp.2008.01.004 18374517

[55] Diot Q Olsen C Kavanagh B Raben D Miften M. Impact of anatomical interventions on the localization of post-prostatectomy cancer patients. Med Phys (2010) 37(2):629–37. 10.1118/1.3284249 20229872

[56] Diot Q Olsen C Kavanagh B Raben D Miften M. Dosimetric effect of online image-guided anatomical interventions for postprostatectomy cancer patients. Int J Radiat Oncol Biol Phys (2011) 79(2):623–32. 10.1016/j.ijrobp.2010.04.020 20643519

[57] Ghilezan MJ Jaffray DA Siewerdsen JH Van Herk M Shetty A Sharpe MB Prostate gland motion assessed with cine-magnetic resonance imaging (cine-MRI). Int J Radiat Oncol Biol Phys (2005) 62(2):406–17. 10.1016/j.ijrobp.2003.10.017 15890582

[58] Padhani AR Khoo VS Suckling J Husband JE Leach MO Dearnaley DP. Evaluating the effect of rectal distension and rectal movement on prostate gland position using cine MRI. Int J Radiat Oncol Biol Phys (1999) 44(3):525–33. 10.1016/S0360-3016(99)00040-1 10348281

[59] de Leon JF Jameson MG Windsor A Cloak K Keats S Vial P Superior target volume and organ stability with the use of endorectal balloons in post-prostatectomy radiotherapy. J Med Imaging Radiat Oncol (2015) 59(4):507–13. 10.1111/1754-9485.12300 25828420

[60] Jameson MG De Leon J Windsor AA Cloak K Keats S Dowling JA Endorectal balloons in the post prostatectomy setting: do gains in stability lead to more predictable dosimetry? Radiother Oncol (2013) 109(3):493–7. 10.1016/j.radonc.2013.08.024 24044793

[61] Smeenk RJ van Lin EN van Kollenburg P McColl GM Kunze-Busch M Kaanders JH. Endorectal balloon reduces anorectal doses in post-prostatectomy intensity-modulated radiotherapy. Radiother Oncol (2011) 101(3):465–70. 10.1016/j.radonc.2011.07.019 21872953

[62] Smeenk RJ Teh BS Butler EB van Lin EN Kaanders JH. Is there a role for endorectal balloons in prostate radiotherapy? A systematic review. Radiother Oncol (2010) 95(3):277–82. 10.1016/j.radonc.2010.04.016 20451274

[63] Ishiyama H Teh BS Blanco AI Paulino AC Mai W-Y Caillouet J Salvage intensity modulated radiotherapy using endorectal balloon after radical prostatectomy: clinical outcomes. Int J Urol (2013) 20(12):1178–83. 10.1111/iju.12145 23573867

[64] Effect of ProSpare (PS), a rectal obturator, on inter- and intrafraction prostate motion and anorectal doses in prostate radiotherapy (RT). J Clin Oncol (2016). Available from: http://meetinglibrary.asco.org/content/158265-172

[65] Murray JR McNair HA Dearnaley DP. Rationale and development of image-guided intensity-modulated radiotherapy post-prostatectomy: the present standard of care? Cancer Manag Res (2015) 7:331–44. 10.2147/CMAR.S51955 26635484 PMC4646477

[66] Zilli T Benz E Miralbell R [Prostate-rectum spacers: optimization of prostate cancer irradiation]. Cancer Radiothér (2014) 18(3):215–21; quiz 243–4, 247. 10.1016/j.canrad.2014.03.001 24746454

[67] Susil RC McNutt TR DeWeese TL Song D. Effects of prostate-rectum separation on rectal dose from external beam radiotherapy. Int J Radiat Oncol Biol Phys (2010) 76(4):1251–8. 10.1016/j.ijrobp.2009.07.1679 19939577 PMC3115781

[68] Rouzaud M Zilli T Vallée JP Weber DC Miralbell R EP-1261: impact of polyethylen glycol spacer injections on interfraction prostate motion in patients undergoing IGRT. Radiother Oncol (2013) 106:S476–7. 10.1016/S0167-8140(15)33567-2

[69] Picardi C Rouzaud M Kountouri M Lestrade L Vallée JP Caparrotti F Impact of hydrogel spacer injections on interfraction prostate motion during prostate cancer radiotherapy. Acta Oncol (2016) 55(7):834–8. 10.3109/0284186X.2015.1128118 26796870

[70] Juneja P Kneebone A Booth JT Thwaites DI Kaur R Colvill E Prostate motion during radiotherapy of prostate cancer patients with and without application of a hydrogel spacer: a comparative study. Radiat Oncol (2015) 10:215. 10.1186/s13014-015-0526-1 26499473 PMC4619294

[71] Pinkawa M Piroth MD Holy R Escobar-Corral N Caffaro M Djukic V Spacer stability and prostate position variability during radiotherapy for prostate cancer applying a hydrogel to protect the rectal wall. Radiother Oncol (2013) 106(2):220–4. 10.1016/j.radonc.2012.11.010 23333015

[72] Pinkawa M. Current role of spacers for prostate cancer radiotherapy. World J Clin Oncol (2015) 6(6):189–93. 10.5306/wjco.v6.i6.189 26677428 PMC4675900

[73] Mok G Benz E Vallee J-P Miralbell R Zilli T. Optimization of radiation therapy techniques for prostate cancer with prostate-rectum spacers: a systematic review. Int J Radiat Oncol Biol Phys (2014) 90(2):278–88. 10.1016/j.ijrobp.2014.06.044 25304788

[74] Pinkawa M Schubert C Escobar-Corral N Holy R Eble MJ. Application of a hydrogel spacer for postoperative salvage radiotherapy of prostate cancer. Strahlenther Onkol (2015) 191(4):375–9. 10.1007/s00066-014-0769-z 25339311

